# Histone deacetylase inhibitors promote epithelial-mesenchymal transition in Hepatocellular Carcinoma *via* AMPK-FOXO1-ULK1 signaling axis-mediated autophagy

**DOI:** 10.7150/thno.47045

**Published:** 2020-08-13

**Authors:** Qiang Xiao, Hao Liu, Hong-Sheng Wang, Meng-Ting Cao, Xiao-Jun Meng, Ya-Li Xiang, Ya-Qin Zhang, Feng Shu, Qiu-Gui Zhang, Hong Shan, Guan-Min Jiang

**Affiliations:** 1 Department of Clinical Laboratory, The Fifth Affiliated Hospital of Sun Yat-sen University, Zhuhai, Guangdong, China.; 2 Cancer Hospital and Cancer Research Institute, Guangzhou Medical University, Guangzhou, Guangdong, China.; 3 Department of Microbial and Biochemical Pharmacy, School of Pharmaceutical Sciences of Sun Yat-sen University, Guangzhou, Guangdong, China.; 4 Department of Clinical Laboratory, The First People's Hospital of Changde City, Changde, Hunan, China.; 5 Department of Clinical Laboratory, The First Affiliated Hospital of the University of South China, Hengyang, Hunan, China.; 6 Department of Health Management, The Fifth Affiliated Hospital of Sun Yat-sen University, Zhuhai, Guangdong, China.; 7 Guangdong Provincial Key Laboratory of Biomedical Imaging, The Fifth Affiliated Hospital of Sun Yat-sen University, Zhuhai, Guangdong, China.; 8 Central Laboratory, The Fifth Affiliated Hospital of Sun Yat-sen University, Zhuhai, Guangdong, China.

**Keywords:** Histone deacetylase inhibitors, Autophagy, Epithelial-mesenchymal transition, FOXO1 inhibitor, Metastasis

## Abstract

Hepatocellular carcinoma (HCC) is the third most frequent cause of cancer-related deaths globally because of high metastasis and recurrence rates. Elucidating the molecular mechanisms of HCC recurrence and metastasis and developing effective targeted therapies are expected to improve patient survival. The promising anti-cancer agents for the treatment of hematological malignancies, histone deacetylase inhibitors (HDIs), have limited effects against epithelial cell-derived cancers, including HCC, the mechanisms involved have not been elucidated. Herein, we studied the molecular mechanisms underlying HDI-induced epithelial-mesenchymal transition (EMT) involving FOXO1-mediated autophagy.

**Methods:** The biological functions of HDIs in combination with autophagy inhibitors were examined both *in vitro* and *in vivo*. Cell autophagy was assessed using the generation of mRFP-GFP-LC3-expressing cells and fluorescent LC3 puncta analysis, Western blotting, and electron microscopy. An orthotopic hepatoma model was established in mice for the *in vivo* experiments.

**Results:** Our study provided novel mechanistic insights into HDI-induced EMT mediated by the autophagy AMPK-FOXO1-ULK1-Snail signaling axis. We demonstrated that autophagy served as a pro-metastasis mechanism in HDI-treated hepatoma cells. HDIs induced autophagy via a FOXO1-dependent pathway, and FOXO1 inhibition promoted HDI-mediated apoptosis in hepatoma cells. Thus, our findings provided novel insights into the molecular mechanisms underlying HDI-induced EMT involving FOXO1-mediated autophagy and demonstrated that a FOXO1 inhibitor exerted a synergistic effect with an HDI to inhibit cell growth and metastasis *in vitro* and *in vivo*.

**Conclusion:** We demonstrated that HDIs triggers FOXO1-dependent autophagy, which ultimately promotes EMT, limiting the clinical outcome of HDI-based therapies. Our study suggests that the combination of an HDI and a FOXO1 inhibitor is an effective therapeutic strategy for the treatment of HCC.

## Introduction

Hepatocellular carcinoma (HCC) is among the most common cancers in humans and is the third most frequent cause of cancer deaths globally 1. Although hepatic resection, liver transplantation, and chemotherapy are commonly used to improve the outcomes of HCC patients, recurrence and metastasis remain the major obstacles to improving HCC patient survival 2. Therefore, it is imperative to gain mechanistic insight into HCC recurrence/metastasis to improve patient survival through targeted treatments.

Histone deacetylase inhibitors (HDIs) are a class of compounds that interfere with the function of histone deacetylases. HDIs specifically induce cancer cells to differentiate, undergo cell cycle arrest, and initiate apoptosis by altering the expression of genes involved in apoptosis signal transduction pathways or cell cycle modulation 3. Epigenetic therapy using HDIs, including trichostatin A (TSA), suberoylanilide hydroxamic acid (SAHA), and sodium butyrate (NaB), has shown promise in clinical trials for the treatment of human hematological malignancies 4,5. However, HDIs have limited effects in epithelial cell-derived cancers, including HCC 6,7. Recently, we found that HDIs treatment induced the epithelial-mesenchymal transition (EMT) in hepatoma cells, a key step in tumor invasion and metastasis 8. Similarly, pharmacological histone deacetylase (HDAC) inhibition decreased breast cancer cell proliferation and tumor growth, yet substantially increased migration and distant metastasis formation 9. In those studies, a single-agent HDIs treatment led to increased cell migration and tumor metastasis, suggesting caution using HDIs alone in the treatment of several solid tumors. Also, it was unclear which mechanisms were dominant in HDI-induced cell invasion and metastasis. Understanding the molecular mechanisms induced by HDIs, particularly exploring their use in combination with other antitumor agents, would be important for developing more effective treatment strategies.

Autophagy is a highly conserved lysosomal degradation pathway that eliminates protein aggregates and dysfunctional organelles to maintain cellular homeostasis 10. Since it has opposing, context-dependent roles in cancer, interventions to both stimulate and inhibit autophagy have been proposed as cancer therapies 11, 12. In response to stress conditions, such as nutrient deprivation, hypoxia, and therapeutic stress, autophagy is activated and serves as a tumor-protective mechanism against adverse environmental and cellular stresses 13. As an adaptive response to stress, autophagy promotes the survival of dormant tumor cells disseminating them in the circulation 14, 15, and specifically links to different stages of the metastatic cascade, including the secretion of pro-migratory factors, EMT, and anoikis resistance 16-18.

In the present study, we studied the regulatory mechanisms underlying the interplay between metastasis and autophagy induced by HDIs. Our findings demonstrated that autophagy served as a pro-metastasis mechanism in HDI-treated hepatoma cells. Furthermore, HDIs induced autophagy through a Forkhead box O1(FOXO1)-dependent pathway, and inhibition of FOXO1 sensitized hepatoma cells to HDIs and overcame autophagy-mediated metastasis. Thus, our findings provide novel insights into the molecular mechanisms underlying HDI-induced metastasis involving FOXO1-mediated autophagy and suggest that combining an HDI and a FOXO1 inhibitor is a more effective therapeutic paradigm for the treatment of hepatocellular carcinoma.

## Materials and Methods

### Chemicals and reagents

NaB and SAHA were purchased from Sigma Aldrich (Deisenhofen, Germany). The FOXO1 inhibitor AS1842856 (AS) was obtained from Selleck, and the adenosine monophosphate-activated protein kinase (AMPK) inhibitor was purchased from MCE. The monoclonal anti-ubiquitin, anti-acetylation, anti-Snail, anti-Smad2, anti-Smad3, anti-Smad2/3, anti-p-Smad2/3, anti-FOXO1, anti-LC3B, anti-P62, anti-ULK1, anti-p-ULK1, anti-LC3, anti-GAPDH, and anti-vimentin antibodies and the secondary anti-mouse or anti-rabbit antibody conjugated to horseradish peroxidase (HRP) were acquired from Cell Signaling Technology (MA, USA). The secondary anti-rabbit antibody conjugated to fluorescein isothiocyanate (FITC), Protein A/G Sepharose and anti-AMPK were purchased from Santa Cruz Biotechnology (Santa Cruz, CA, USA). Anti-p-AMPK and anti-FN were acquired from Abcam. A Millicell chamber (8 mm) was purchased from Millipore (BD Biosciences, USA). The Annexin V, FITC apoptosis detection Kit S was obtained from Japan Tongren Chemical. SYBR Premix ExTaq II was a product of TaKaRa (TBI, Japan). siRNA FOXO1 and siRNA ULK1 were purchased from RIBO, and Lipofectamine 3000 was purchased from Invitrogen (Carlsbad, CA, USA). 4,6-diamidino-2-phenylindole (DAPI) dye was obtained from FUDE company.

### Cytotoxicity assay

The cytotoxicity of SAHA and NaB toward cultured cells was assessed using the MTT [3-(4,5-dimethylthiazol-2yl)-2,5-diphenyltetrazolium bromide] assay (Sigma Chemical Co). Cells were seeded in 96-well microplates at a density of 1×10^4^ cells per well and incubated for 24 h. The cells were then treated with the selected concentrations of SAHA and NaB for 24 h. Cells in culture medium alone served as the untreated control. The MTT reagent (5 mg/mL in distilled water) was prepared immediately prior to use. After removing the incubation medium from the wells, the cells were washed with PBS, and 10 μL of MTT reagent was added. After incubation for 4 h at 37 °C, MTT reagent in 100 μL of dimethylsulfoxide (DMSO) was added to each well. The surviving cells were then detected by measuring absorbance at 570 nm using a plate reader. Cell viability was expressed as a percentage of the values obtained for the controls.

### Gene expression profiling

HepG2 and PLC cell lines were treated with or without 3 mM NaB or 3 μM SAHA for 24 h, and total RNA was extracted using TRIzol reagent (Takara, Cat. #9108). RNA quantity and integrity were assessed using NanoDrop ND-2000 (Thermo Scientific) and the Agilent Bioanalyzer 2100 (Agilent Technologies). The gene expression profiling was performed by Shanghai Oebiotech Corporation using the Agilent SurePrint G3 Human Gene Expression v3 Panel (Agilent, CA, USA). All data were analyzed according to the manufacturer's protocol. Genes with a fold change > 2 or < -2 and an adjusted P < 0.05 were considered differentially expressed.

### Western blotting analysis

Cells were lysed in cell lysis buffer, and lysates were cleared by centrifugation and denatured by boiling in the Laemmli buffer. Equal amounts of protein samples were separated on 12% sodium dodecyl sulfate (SDS) polyacrylamide gels and electrophoretically transferred to nitrocellulose membranes. Following blocking with 5% nonfat milk at room temperature for 2 h, the membranes were incubated with the primary antibody at a 1:1000 dilution overnight at 4 °C and then incubated with an HRP-conjugated secondary antibody at 1: 5000 dilution for 1 h at room temperature. Specific immune complexes were detected using Chemiluminescence Reagent Plus (Life Science, Inc. Boston, MA, USA) for Western blotting.

### Wound-healing assay

HepG2 and PLC cells (4×10^5^) were cultured on 6-well plates. After 24 h, the cells covered the entire bottom of the well. A defined scratch was applied on the well bottom, which detached cells within a corridor. Cells were treated with or without different drugs for 24 h. Over a period of 24 h, the percentage recovering of the scratch wound was monitored.

### Cell invasion assay

The cell invasion assay was performed as follows: First, 6.5 mm diameter polycarbonate filters (8 mm pore size) were coated with Matrigel™, dried, and reconstituted at 37 °C with appropriate DMEM before use. Then, 1×10^5^ HepG2 or PLC cells per chamber were added to the upper chamber in DMEM containing 1% FCS, while the lower chamber contained DMEM with 10% FCS. Cells were treated with or without different drugs for 24 h, and the drug concentration was the same in the lower and upper chambers. After 24 h of incubation at 37 °C in a CO_2_ incubator, the number of cells that had migrated through the pores of the filter and into the lower chamber was counted under a phase-contrast microscope.

### Immunoprecipitation

To assess the ubiquitination and acetylation of FOXO1, cells were treated with NaB or SAHA for 2 h, washed twice with ice-cold phosphate-buffered saline (PBS) and harvested at 4 °C in immunoprecipitation lysis buffer [50 mmol/L HEPES, pH 7.5, 150 mmol/L NaCl, 2 mmol/L EDTA, 0.5% NP-40,10% glycerol, 1 mmol/L NaF, 1 mmol/L Na3VO4, 1 mmol/L dithiothreitol, 1 mmol/L 4-(2-aminoethyl) benzenesulfonylfluoride, leupeptin (1 mg/mL), aprotinin (1 mg/mL), and pepstatin (1 mg/mL)]. Equal amounts of protein were immunoprecipitated using anti-FOXO1 antibody, and the immune complexes were bound to protein A/G Sepharose. The beads were washed with lysis buffer and subjected to Western blotting with anti-ubiquitin or anti-acetylate antibody.

### Flow cytometry for apoptosis

HepG2 and PLC cells were treated with or without 0.5 μmol/L AS for 24 h. The cells were treated with SAHA or NaB for 24 h and washed twice with precooled PBS. Subsequently, 500 μL of binding buffer was added to the cell suspension, followed by 5 μL of Annexin V and FITC. Finally, 5 μL of propidium iodide (PI) was added to the samples, incubated at room temperature for 15 min in the dark, and analyzed immediately using FL1 (Em: 525 nm) and FL3 (Em: 670 nm) by flow cytometry (Beckman-Coulter, Miami, USA).

### Electrophoresis mobility shift assay (EMSA)

Nuclear extracts from cells treated with or without 3 mM NaB or 3 µM SAHA for 1 h were used in EMSA with an oligonucleotide probe, which was the sequence from the Snail promotor containing the Smads binding site (CAGAA/C). The coding strand of the oligonucleotide probe was 5'-CCAGGGGGCGTCAGAAGCGCTCAGACCACCGGGCGC-3'. Antibodies used for supershifting the complex were purchased from Santa Cruz (Santa Cruz, CA).

### Quantitative real-time polymerase chain reaction (qRT-PCR)

Cells (2×10^5^) were plated in 6-well plates, and after treatment with drugs, the cells were washed twice with ice-cold PBS. Total mRNA was extracted with TRIZOL reagent. The first strand of cDNA was generated from 2 μg of total RNA using oligo-dT primer and Superscript II Reverse Transcriptase (GIBCO BRL, Grand Island, NY, USA). qRT- PCR was run on an iCycler (Bio-Rad, Hercules, CA, USA) using validated primers and SYBR Premix Ex Taq II (Takara, Japan) for detection. When the fluorescence first reached a preset threshold (Ct), the cycle number allowed the quantification of the specific template concentration. Transcripts of the housekeeping gene glyceraldehyde 3-phosphate dehydrogenase (GAPDH) in the same incubations were used for internal normalization. The primer pairs used in the subsequent qRT-PCR reactions were as follows: Snail, forward 5′-GACCACTATGCCGCGCTCTT-3′ and reverse 5′-TCGCTGTAGTTAGGCTTCCGATT-3′; ULK1, forward 5′-AGCCATTTCCTGGAAGTCGTA-3′ and reverse 5′-CTGAACCGCTCCAAGAAGAAC-3′; FOXO1, forward 5′-CTTCAAGGATAAGGGCGACA-3′ and reverse 5′-ATTTAAGCGGTGTTAGACAG-3′; GAPDH, forward 5′-GGACCTGACCTGCCGTCTAG-3′ and reverse 5′-AGTTCCCGTAGGACCCGATG-3′.

### Generation of mRFP-GFP-LC3-expressing cells and fluorescent LC3 puncta analysis

Cells were transfected with the mRFP-GFP-LC3 plasmid (a gift from the University of Texas Southwestern Medical Center, Dr. Joseph A) for 24 h followed by transfection with si-FOXO1 or pretreatment with inhibitors of FOXO1 for 24 h, and subsequently treated with SAHA or NaB for 12 h. The RFP and GFP fluorescence labeled the intracellular distribution of LC3 protein, which was captured by confocal laser scanning microscopy (Olympus, Japan) of the red and green spot colocalization. Macros and Image J programs were used to quantify LC3 spots, and the amount of LC3 of each cell was calculated. The average number of spots was determined; the GFP^+^ RFP^+^ puncta were yellow, and the GFP^-^RFP^+^ puncta were red. The experiment was repeated three times.

### Confocal microscopy for Smad2/3

Cells were grown on chamber slides. After treatment with or without 0.5 µmol/L AS for 24 h, the cells were stimulated with NaB or SAHA for 30 min, fixed in 4% paraformaldehyde for 30 min, blocked with goat serum for 30 min at 37 °C, and then incubated with the anti-Smad2/3 antibody at 1: 100 for 1 h at 37 °C. The slides were washed with PBS and incubated with the secondary anti-mouse antibody conjugated to FITC at 1: 1000 for 45 min at 37 °C. After washing with PBS, the cells were incubated with DAPI (10 mg/mL) for 10 min to visualize cell nuclei. Samples were examined with confocal laser scanning microscopy (Olympus, Japan) to analyze the nuclear translocation of Smad2/3.

### *In vitro* gene silencing

The Validated Stealth TM Negative Control and double-stranded FOXO1-specific, Unc-51-like kinase 1(ULK1)-specific small interference RNA (siRNA) oligonucleotides were purchased from Guangzhou Ruibo Biotechnology Co., Ltd. The target sequence of si-ULK1 was TCACTGACCTGCTCCTTAA. The target sequence of si-FOXO1 was CCAGATGCCTATACAAACA. Cells were transfected using Lipofectamine 3000 reagent according to the manufacturer's instructions and incubated for 48 h before harvest.

### Electron microscopy for detection of autophagosomes

Two liver cancer cell lines, HepG2 and PLC, were transfected with a FOXO1 inhibitor for 24 h. Then, the cells were treated with SAHA or NaB for 12 h and then immediately treated with 2% glutaraldehyde and 2% paraformaldehyde. The cells were fixed in sodium cacodylate buffer (pH 7.2) for 3 h at room temperature and then fixed at 4 ° C for 16 h. Postfixation was carried out for 1 h at room temperature in 1% osmium tetroxide in sodium dimethyl citrate buffer. After dehydration by a graded series of ethanol, all samples were kept in epoxy resin (Agar Scientific, AGR 1043) for 16 h prior to embedding. Ultrathin sections were collected on a Formvar-coated 200 mesh or single hole copper grid (Agar Scientific, AGS162) and stained with 2% uranyl acetate in 50% ethanol and lead citrate for 10 min each. Electron microscopic images were obtained using an HT7700 transmission electron microscope (Hitachi, Japan).

### Construction of orthotopic hepatoma model in mouse

HepG2-luc cells (luciferase gene-labeled human hepatoma cell line HepG2) in the logarithmic growth phase cultured under standard conditions were digested and centrifuged, and 5×10^6^ cells obtained were dissolved in 0.1 mL of DMEM culture medium. Next, the SPF thymus Balb/C nude mice were anesthetized with 3% pentobarbital sodium. After the anesthesia took effect, the liver was exposed by open surgery. The cells were injected into the liver of the nude mice with a 1 mL insulin injection needle, and the wound was sutured with 5-0 silk thread. The mouse was placed on a heating pad and allowed a normal diet after reviving. Live imaging was then performed on nude mice after tumor formation. The mice were anesthetized with 3% pentobarbital sodium before imaging and then intraperitoneally injected with 3 mg of the substrate luciferin. The substrate was injected for 10 min and then subjected to *in vivo* imaging. Finally, the animals were sacrificed in accordance with ethical procedures, and the mouse liver tissues were used for immunohistochemical staining to analyze the markers of autophagy and EMT.

### Statistical analyses

All values were reported as the mean ± SEM of three independent experiments unless otherwise specified. The data were analyzed by two-tailed unpaired Student's t-test between two groups and by one-way analysis of variance (ANOVA) followed by Bonferroni test for multiple comparisons. These analyses were performed using GraphPad Prism Software Version 5.0 (GraphPad Software, Inc. La Jolla, CA, USA). *p* < 0.05 was considered significant.

## Results

### HDIs induce EMT, invasion, and metastasis

**Figure 1:**
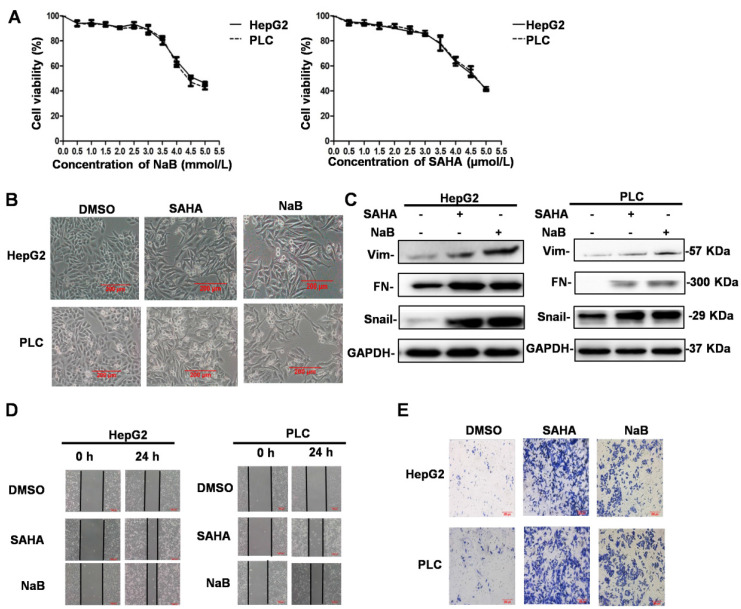
** HDI-induced EMT, invasion, and metastasis in hepatoma cells.** (**A**) Cells were incubated in the presence of different concentrations of HDIs for 24 h, and the cytotoxicity of HDIs in hepatoma cells was determined by the MTT enzyme assay. (**B-E**) HepG2 and PLC cells were treated with or without 3 mM NaB (dissolved in water) or 3 µM SAHA (dissolved in DMSO) for 24 h, and phenotypic changes of EMT were detected using a phase-contrast microscope (B). Protein expression of vimentin, fibronectin, and Snail was detected by Western blotting (C). Invasion and metastasis of hepatoma cells were detected by wound-healing and cell invasion assays, respectively (D-E). Scale bar, 200 µm. Similar results were obtained in three independent experiments.

An MTT enzyme assay was used to determine the cytotoxicity of HDIs (NaB and SAHA) in human liver cancer cell lines, HepG2 and PLC. As shown in Figure 1A, NaB and SAHA suppressed the proliferation of cells in a concentration-dependent manner. At 3 μM SAHA and 3 mM NaB, about 90% of cells survived, whereas the cells died when exposed to higher concentrations (Figure 1A). Therefore, in subsequent experiments, we used 3 mM NaB and 3 μM SAHA, which did not affect the survival rate of the hepatoma cells significantly. To determine the effect of HDIs on EMT, hepatoma cell lines were treated with or without SAHA or NaB for 24 h. Subsequently, phenotypic changes were detected under a phase-contrast microscope, and the protein expression of the EMT molecular markers vimentin, fibronectin, and Snail were detected by Western blotting. After treatment with NaB or SAHA, HepG2 and PLC cells became scattered and adopted a typical fibroblast-like morphology of mesenchymal cells (Figure 1B), and the protein expression levels of vimentin, fibronectin, and Snail were upregulated significantly (Figure 1C). These results suggested that HDIs could induce EMT in hepatoma cells, which is consistent with our previous studies that Snail plays a key role in HDI-induced EMT 8. To detect changes in the invasive and metastatic ability after EMT, the hepatoma cells were treated with or without SAHA or NaB for 24 h, and invasion and metastasis were assessed by wound-healing and cell invasion assays, respectively. The scratch wound of cells treated with SAHA or NaB became significantly narrower than the control (Figure 1D). Also, after treatment with SAHA or NaB for 24 h, the number of cells passing through the pores of the filter and into the lower chamber increased significantly compared with the control (Figure 1E). These findings demonstrated that HDIs could induce EMT and trigger the invasion and metastasis of hepatoma cells.

### HDIs induce autophagy

**Figure 2:**
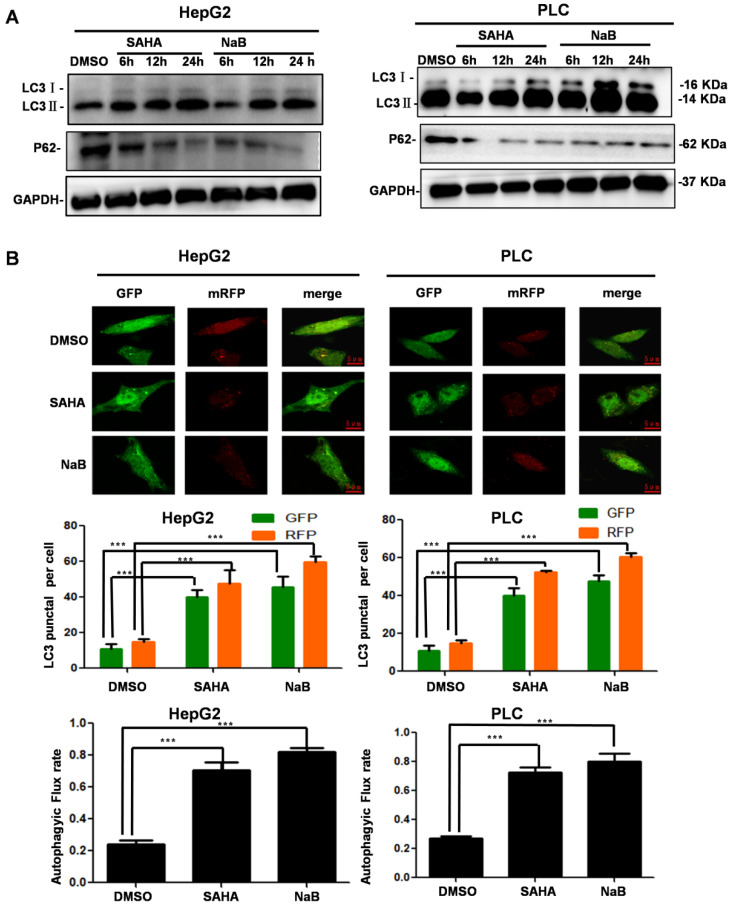
** HDI-induced autophagy in hepatoma cells.** (**A**) HepG2 and PLC cells were treated with or without 3 mM NaB or 3 µM SAHA for 6, 12, and 24 h, and markers of autophagy (LC3-I, LC3-II, P62) were detected by Western blotting. (**B**) The mRFP-eGFP-LC3 plasmid was transfected into HepG2 and PLC cells for 24 h, and the cells were treated with or without 3 mM NaB or 3 µM SAHA for 12 h; fluorescent were counted under confocal microscopy, scale bar, 5 µm. Similar results were obtained in three independent experiments. ***P<0.0001.

We treated hepatoma cells with HDIs for 6, 12, and 24 h and investigated the effect on autophagy. After treatment with SAHA or NaB for 12 h, LC3-II accumulation was observed, and P62 expression was downregulated significantly, suggesting that HDIs induced autophagy (Figure 2A). To further determine the role of HDIs in autophagy, hepatoma cells were transfected with the mRFP-GFP-LC3 plasmid for 24 h, then treated with SAHA or NaB for 12 h. HDIs significantly increased the autophagic flux level as detected by confocal microscopy and GFP-LC3 puncta representing autophagic vacuoles (Figure 2B). Collectively, these results demonstrated that HDIs could induce autophagy in hepatoma cells.

### Role of FOXO1 and ULK1 in regulating autophagy and EMT

**Figure 3:**
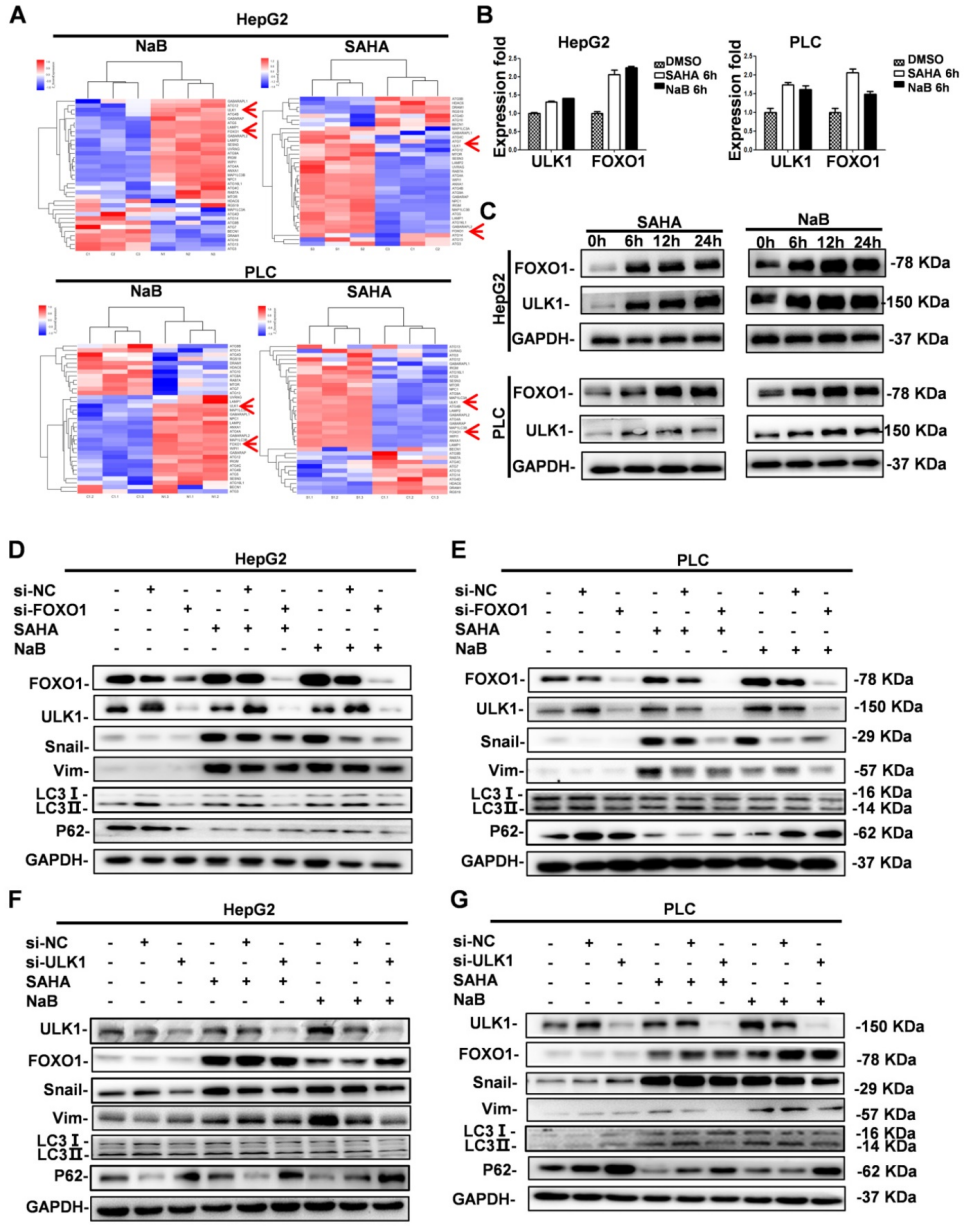
** Role of FOXO1 and ULK1 in regulating autophagy and EMT in hepatoma cells.** (**A-B**) HepG2 and PLC cells were treated with or without 3 mM NaB or 3 µM SAHA for 6 h; the expression of autophagy-related genes was assessed using gene chip technology (A), mRNA levels of FOXO1 and ULK1 were detected by real-time PCR (B). (**C**) HepG2 and PLC cells were treated with or without 3 mM NaB or 3 µM SAHA for 6 h, 12 h, and 24 h, and the protein expression levels of FOXO1 and ULK1 were detected by Western blotting. Similar results were obtained in three independent experiments. (D-G) HepG2 and PLC cells were transfected with or without FOXO1 siRNA (**D-E**) or ULK1 siRNA (**F-G**) for 24 h; the cells were treated with or without 3 mM NaB or 3 µM SAHA for 24 h, and the expression levels of Snail, vimentin, LC3I, LC3II and P62 were detected by Western blotting. Similar results were obtained in three independent experiments.

Previously, a distinct function of FOXO1 in HDI-mediated autophagy in human cancer cells was reported, suggesting the possibility of a novel therapeutic strategy by combining HDIs and autophagy inhibitors in cancer therapy 19. However, the downstream regulatory mechanism of FOXO1 in HDI-induced autophagy was unclear. Therefore, we first investigated FOXO1 expression in HDI-treated hepatoma cells by gene expression profiling. Analysis of autophagy-related gene expression showed significant upregulation of FOXO1 and ULK1 (Figure 3A). To further determine the key role of FOXO1 and ULK1 in HDI-induced autophagy, qRT-PCR and Western blotting were performed. The results demonstrated that HDIs upregulated FOXO1 and ULK1 in hepatoma cells in a time-dependent manner (Figure 3B & C), indicating their key regulatory role in HDI-induced autophagy.

In our previous study, we found that HDIs promoted the expression of Snail, and Snail-induced EMT was critical for HDI-initiated invasion and metastasis 8. The present study found that HDIs could induce autophagy in hepatoma cells by upregulating FOXO1 and ULK1. To determine the causal relationship between FOXO1 and ULK1 and their role in EMT and autophagy, the expression of FOXO1 and ULK1 was silenced by transfection with siRNAs, and the cells were subsequently treated with 3 μM SAHA or 3 mM NaB. The results showed decreased expression of EMT molecular markers vimentin and Snail and also of LC3-II but an upregulated expression of the autophagy molecular marker P62 that were comparable to the control group. Furthermore, when FOXO1 expression was silenced, ULK1 expression was downregulated, but when ULK1 expression was silenced, FOXO1 expression was not affected (Figure 3D-G). Therefore, the results demonstrated that FOXO1 and ULK1 played a key role in HDI-induced EMT and autophagy, and FOXO1 regulated ULK1 expression.

### FOXO1 inhibitor antagonizes HDI-induced autophagy and EMT

HDIs are known to induce apoptosis via multiple mechanisms, such as increasing the expression of BIM and other related genes 20. Studies have shown that EMT is a critical contributor to the invasion and metastasis of epithelial-derived cancers 21, 22, and that autophagy serves as an important cell survival mechanism by inhibiting apoptosis 19. Because we demonstrated that HDIs were able to induce EMT and autophagy in which FOXO1 played a key role, a FOXO1 inhibitor, when combined with HDIs, might be a promising therapeutic approach for cancer.

**Figure 4:**
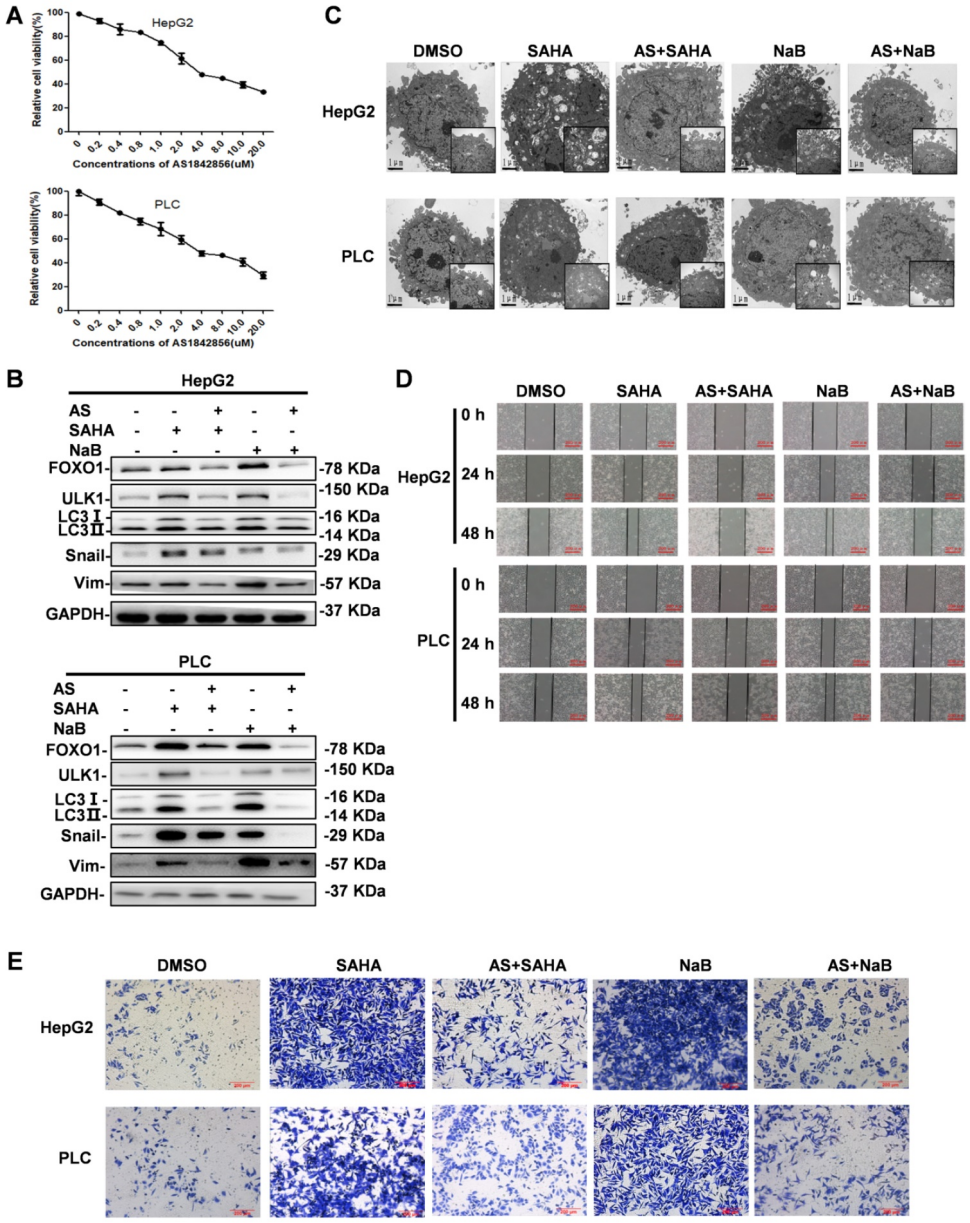
** FOXO1 inhibitor antagonizes HDI-induced autophagy and EMT in hepatoma cells.** (**A**) HepG2 and PLC cells were incubated in the presence of different concentrations of the FOXO1 inhibitor AS for 24 h, and the cytotoxicity of AS in hepatoma cells was determined by the MTT assay. (**B**) HepG2 and PLC cells were pretreated with or without 1 µM AS for 24 h and then treated with or without 3 mM NaB or 3 µM SAHA for 24 h, and the protein expression levels of FOXO1, ULK1, LC3-I, LC3-II, Snail, and vimentin were detected by Western blotting. (**C-E**) HepG2 and PLC cells were pretreated with or without 1 µM AS for 24 h and then with or without 3 mM NaB or 3 µM SAHA for different times; the morphological structure of autophagy were examined by transmission electron microscopy, scale bar, 1 µm (C) Invasion and metastasis were detected by wound-healing assay (D) and (E) cell invasion assay. Similar results were obtained in three independent experiments.

To determine the effect of the FOXO1 inhibitor AS on autophagy and EMT, its cytotoxicity in hepatoma cells was determined by the MTT assay. Based on the results displayed in Figure 4A, showing that AS induced cell death in a dose-dependent manner, we selected 1 µM AS for subsequent *in vitro* experiments. Cells were pretreated with AS for 24 h followed by treatment with NaB or SAHA for 24 h. The results showed that AS inhibited the expression of HDI-induced FOXO1. Western blotting revealed that AS also inhibited autophagy markers ULK1 and LC3-II and the EMT markers Snail and vimentin, which were upregulated by HDIs (Figure 4B). The effect of AS on autophagy was also examined by electron microscopy, demonstrating that when FOXO1 was inhibited by AS, the autophagosomes that had increased after HDI treatment reduced to normal levels (Figure 4C). Furthermore, wound-healing and cell invasion assays indicated that AS could inhibit the invasion and metastasis of hepatoma cells triggered by HDI-induced EMT (Figure 4D & E). These results indicated that the FOXO1 inhibitor AS could antagonize HDI-induced autophagy and EMT in hepatoma cells, further asserting the benefits of combining AS with HDIs for cancer therapy.

### FOXO1 inhibitor represses HDI-induced expression of Snail by inhibiting Smad2/3 phosphorylation and nuclear translocation

**Figure 5:**
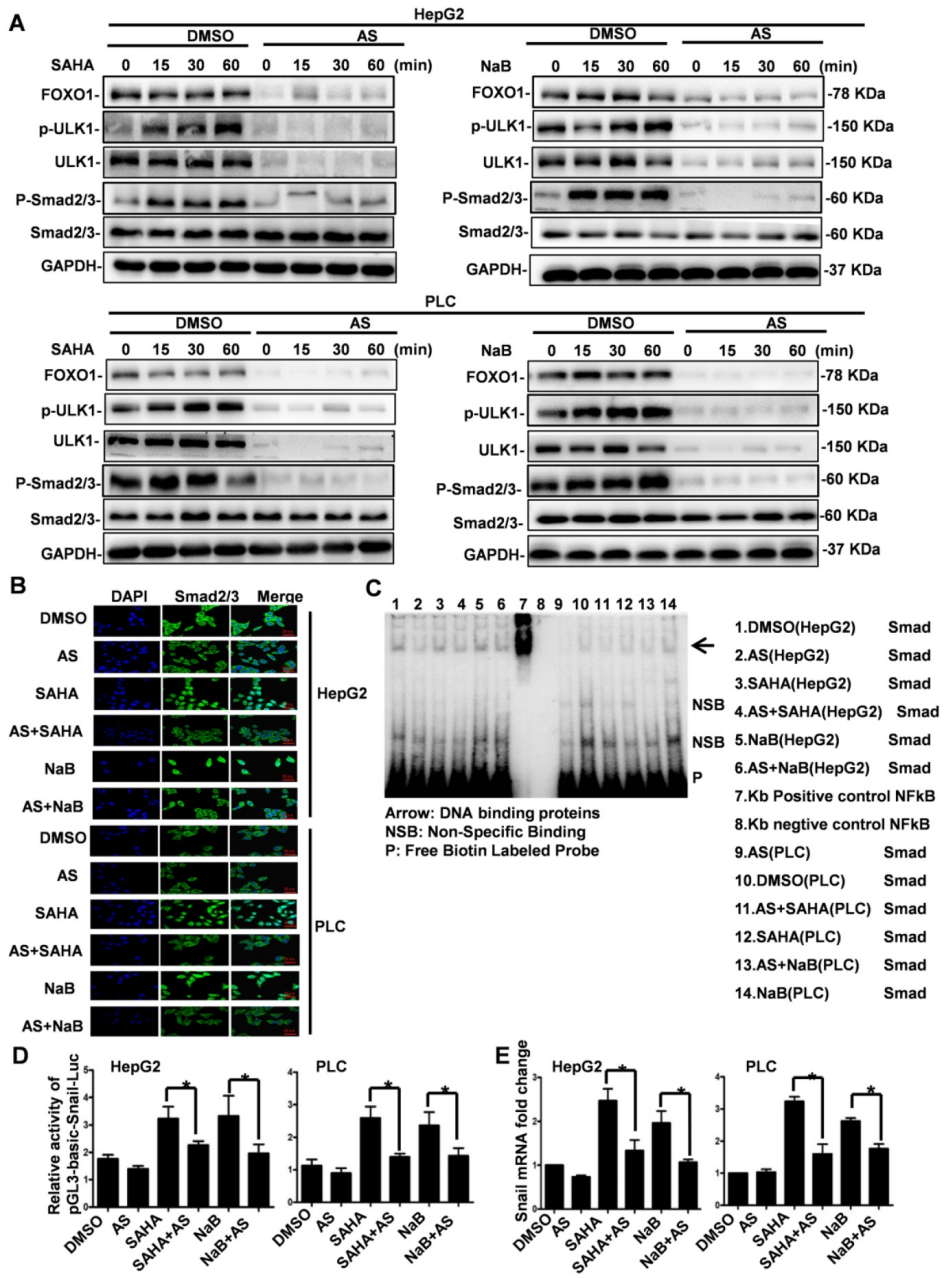
** FOXO1 inhibitor represses HDI-induced expression of Snail by inhibiting Smad2/3 phosphorylation and nuclear translocation.** (**A**) Cells were pretreated with or without 1 µM AS for 24 h followed by treatment with or without 3 mM NaB or 3 µM SAHA for 15, 30 min, and 1 h. The phosphorylation of ULK1 and Smad2/3 was detected by Western blotting. GAPDH served as the loading control. Similar results were obtained in three independent experiments. (**B**) Cells were pretreated with or without 1 µM AS for 24 h and then with or without 3 mM NaB or 3 µM SAHA for 30 min, and immunofluorescence and confocal microscopy were used to detect Smad2/3, scale bar, 20 µm. (**C**) Cells were pretreated with or without 1 µM AS for 24 h and then with or without 3 mM NaB or 3 µM SAHA for 1 h, and the binding of Smads to the Snail promoter was detected by EMSA. (**D**) Cells were transfected with pGL3-Basic-Snail-luc reporter plasmid, pretreated with or without 1 µM AS for 24 h, and then with or without 3 mM NaB or 3 µM SAHA for 24 h. Luminescence was measured using a luminometer. pRL-TK plasmids served to correct for transfection efficiency. The results are expressed as the ratios between the activity of the reporter plasmid and pRL-TK, *p < 0.01. (**E**) Cells were pretreated with or without 1 µM AS for 24 h and then with or without 3 mM NaB or 3 µM SAHA for 24 h. Snail mRNA was detected by qRT-PCR.

We have previously shown that HDI-triggered EMT, invasion, and metastasis of hepatoma cells are mediated through inducing Snail's transcriptional expression via the Smad2/3 pathway 8. Our present study indicated that HDIs induce autophagy via the FOXO1-ULK1 pathway, but whether HDI- regulated Snail expression through the Smad2/3 pathway is involved still needed to be clarified. To determine FOXO1's role in HDI-induced Snail expression, cells were first treated with AS for 24 h and then with NaB or SAHA for 15, 30 min, and 1 h. As shown in Figure 5A, HDIs promoted the phosphorylation of ULK1 and Smad2/3 significantly. When FOXO1 was inhibited by AS, ULK1 together with its phosphorylated form and Smad2/3 were also considerably inhibited, but Smad2/3 expression was not affected. These results demonstrated that AS inhibited HDI-induced phosphorylation of Smad2/3 and ULK1 (Figure 5A).

To further examine whether AS repressed HDI-induced Smad2/3 nuclear translocation, immunofluorescence and confocal microscopy were used. The results demonstrated that Smad2/3 were localized exclusively in the cytoplasm in untreated cells, while HDI treatment for 30 min induced a significant nuclear translocation of Smad2/3. The inhibition of FOXO1 by AS also blocked the HDI-induced nuclear translocation of Smad2/3 (Figure 5B). Following translocation to the nucleus, Smad2/3 bind to gene promoters to carry out their biological functions. It has been reported that Smads bind to the gene promoter sequence CAGAA/C 23. We found many consensus sequences of CAGAA/C in the Snail promoter (422-436). EMSA results confirmed that the FOXO1 inhibitor AS inhibited the binding of Smads to the Snail promoter facilitated by HDIs (Figure 5C). The dual-Glo-luciferase analysis revealed that AS significantly inhibited the activity of pGL3-Basic-Snail-luc, which was enhanced by HDIs in hepatoma cells (Figure 5D). Also, qRT-PCR demonstrated that AS inhibited the transcriptional expression of Snail promoted by HDIs. The fold-increase of Snail mRNA in the HDI-treated cells was more than double of that in the control group, and when FOXO1 was inhibited by AS, Snail mRNA was returned to normal levels (Figure 5E). Collectively, our results indicated that the FOXO1 inhibitor AS repressed the HDI-induced expression of Snail by inhibiting Smad2/3 phosphorylation and nuclear translocation, preventing the binding of Smad2/3 to the promoter and thus blocking the transcription of Snail. These findings provide new insight into the molecular details of HDI-induced EMT in hepatoma cells.

### FOXO1 inhibitor combined with HDIs inhibits proliferation and promotes apoptosis

**Figure 6:**
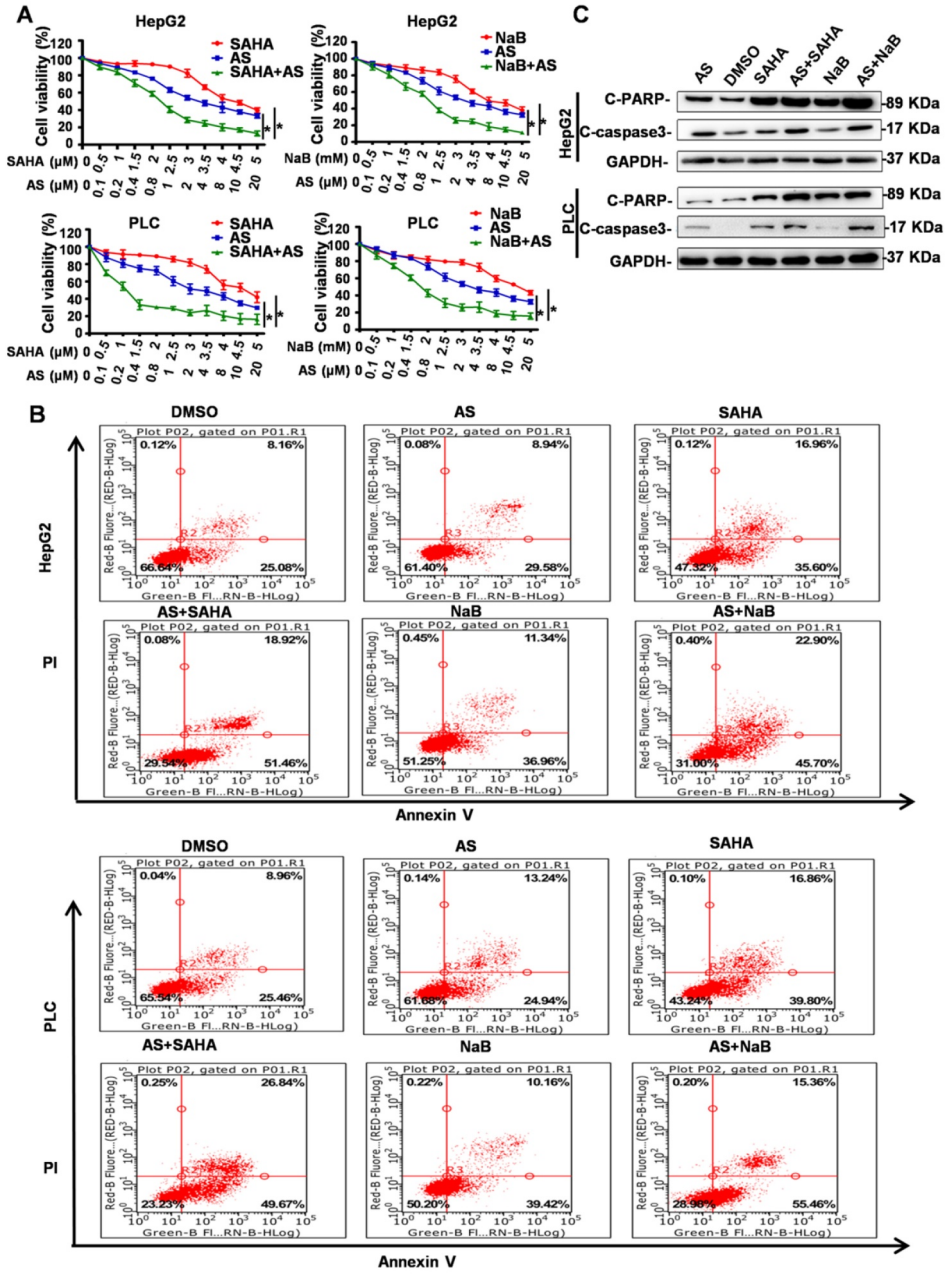
** FOXO1 inhibitor combined with HDIs inhibits proliferation and promotes apoptosis in hepatoma cells.** (**A**) HepG2 and PLC cells were treated with NaB or SAHA and AS at indicated concentrations. Cell viability was determined by the MTT enzyme assay, *p < 0.01. (**B-C**) HepG2 and PLC cells were pretreated with or without 1 µM AS for 24 h and then with or without 3 mM NaB or 3 µM SAHA for 24 h; (B) cell apoptosis was calculated by flow cytometry, (C) Expression of Cleaved PARP and Cleaved Caspase-3 was measured by Western blotting. Similar results were obtained in three independent experiments.

HDIs were reported to exert their antitumor activities by inducing cell cycle arrest, differentiation, and apoptosis. Because the FOXO1 inhibitor AS can inhibit the side effects of EMT and autophagy induced by HDIs, we investigated whether AS combined with HDIs could enhance the function of HDIs in inhibiting proliferation and promoting apoptosis. To determine the effect of AS combined with HDIs on hepatoma cell proliferation, HepG2 and PLC cell lines were pretreated with AS for 24 h followed by treatment with NaB or SAHA for 24 h, and cell proliferation was evaluated using the MTT assay. The results showed that combinations of HDIs and AS compared to either agent alone exhibited a more potent activity to induce anti-proliferative effects in HepG2 and PLC cells (Figure 6A). These data suggested that HDIs in combination with AS exerted a synergistic effect on growth inhibition in hepatoma cells.

Flow cytometry and Western blotting were used to determine the effect of AS combined with HDIs on hepatoma cell apoptosis. As shown in Figure 6B, flow cytometry results demonstrated that the percent of apoptotic cells (Annexin V^+^) in the control group and the groups treated with DMSO or AS alone was below 40%, while cells treated with HDIs exhibited approximately 50% apoptotic cells. When the cells were treated with AS and HDIs, the percent of apoptotic cells was 68.6%-76.51% (Figure 6B), indicating that the combination could enhance HDI-induced apoptosis in hepatoma cells. Further, Western blotting demonstrated that when cells were treated with AS together with HDIs, expression of the apoptotic markers, C-PARP and C-caspase, increased significantly compared with cells treated with HDIs alone (Figure 6C). These results indicated that the FOXO1 inhibitor AS enhanced the function of HDIs in inhibiting proliferation and promoting apoptosis in hepatoma cells, and the combination could be an effective method for treating hepatic carcinoma.

### HDIs upregulate FOXO1 by activating AMPK and inhibiting the degradation of FOXO1

**Figure 7:**
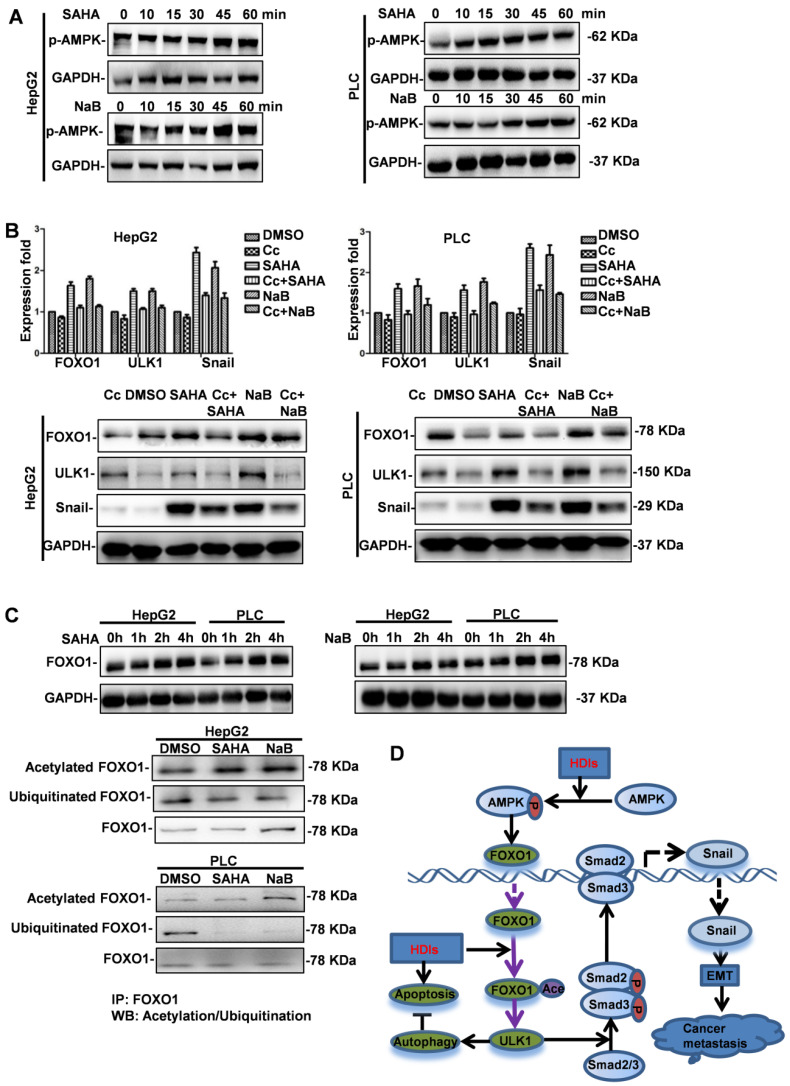
** HDIs upregulate FOXO1 by activating AMPK and inhibiting the degradation of FOXO1.** (**A**) HepG2 and PLC cells were treated with or without 3 mM NaB or 3 µM SAHA for 10, 15, 30, 45, and 60 min. AMPK phosphorylation was detected by Western blotting. GAPDH served as a loading control. Similar results were obtained in three independent experiments. (**B**) Cells were pretreated with or without 4 µM of the AMPK inhibitor Compound C (Cc) for 2 h, then treated with or without 3 mM NaB or 3 µM SAHA for 12 h, and the expression of FOXO1, ULK1 and Snail was detected by qRT-PCR and Western blotting. GAPDH served as the loading control. Similar results were obtained in three independent experiments. (**C**) Cells were treated with or without 3 mM NaB or 3 µM SAHA for 1, 2, and 4 h, and FOXO1 expression was detected by Western blotting. GAPDH served as the loading control. Subsequently, cells were treated with or without 3 mM NaB or 3 µM SAHA for 2 h, and the total proteins in the cell lysate were subjected to immunoprecipitation with an anti-FOXO1 antibody. The acetylation or ubiquitination of FOXO1 levels in the immune complex was detected by Western blotting with an anti-acetylate or an anti-ubiquitin antibody; FOXO1 served as the loading control. Similar results were obtained in three independent experiments. (**D**) A proposed model to illustrate the mechanism of HDI induction of FOXO1 expression and the promotion of hepatoma cell EMT and autophagy. **→**direct stimulation; ─**|**direct inhibition.

Although our results demonstrated that FOXO1 played a key role in HDI-induced EMT and autophagy, the mechanism of upregulated FOXO1 expression by HDIs was not known. It was reported that PI3K/AKT/mTOR, a key pathway in regulating autophagy, was regulated by AMPK 24. Therefore, we investigated the role of AMPK in HDI-induced FOXO1 expression. Cells were treated with SAHA or NaB for 10, 15, 30, 45 min, and 1 h, and AMPK phosphorylation was analyzed by Western blotting. The results demonstrated that HDIs promoted rapid phosphorylation of AMPK in a time-dependent manner (Figure 7A). To further elucidate the role of AMPK in regulating autophagy and EMT, cells were pretreated with or without the AMPK inhibitor Compound C (Cc) for 2 h. Subsequently, the cells were treated with NaB or SAHA for 12 h, and the expression levels of FOXO1, ULK1 and Snail were determined by qRT-PCR and Western blotting. The results demonstrated that HDIs significantly upregulated FOXO1, ULK1, and Snail expression, and when AMPK was inhibited by Cc, a significant downregulation of FOXO1, ULK1 and Snail was observed both at mRNA and protein levels (Figure 7B). These results indicated that HDIs upregulated FOXO1 expression via AMPK. Furthermore, the observation that FOXO1 protein expression was increased when cells were treated with HDIs for 2h (Figure 7C) indicated that post-transcriptional regulation might be involved. The ubiquitin-proteasome is a protein degradation pathway involving the covalent modification of cellular proteasome substrates with poly-ubiquitin chains, which targets them for further degradation. To determine whether the proteasome machinery was involved in the HDI-induced upregulation of FOXO1, cells were treated with NaB or SAHA for 2 h. Subsequently, total proteins in the cell lysates were subjected to immunoprecipitation with an anti-FOXO1 antibody. The acetylation or ubiquitination of FOXO1 levels in the immune complex was detected by Western blotting with an anti-acetylate or an anti-ubiquitin antibody. The results demonstrated that FOXO1 acetylation was considerably enhanced, while FOXO1 ubiquitination was highly suppressed in cells treated with HDIs for 2 h compared with control cells (Figure 7C). These results provided evidence that HDIs upregulated FOXO1 by promoting its acetylation and inhibiting ubiquitination and degradation. Taken together, our findings demonstrated that HDIs upregulated FOXO1 levels by enhanced transcription via the AMPK pathway and promoted its stabilization by inhibiting intracellular protein degradation (Figure 7D).

### HDIs combined with a FOXO1 inhibitor elicit a protective antitumor response

**Figure 8:**
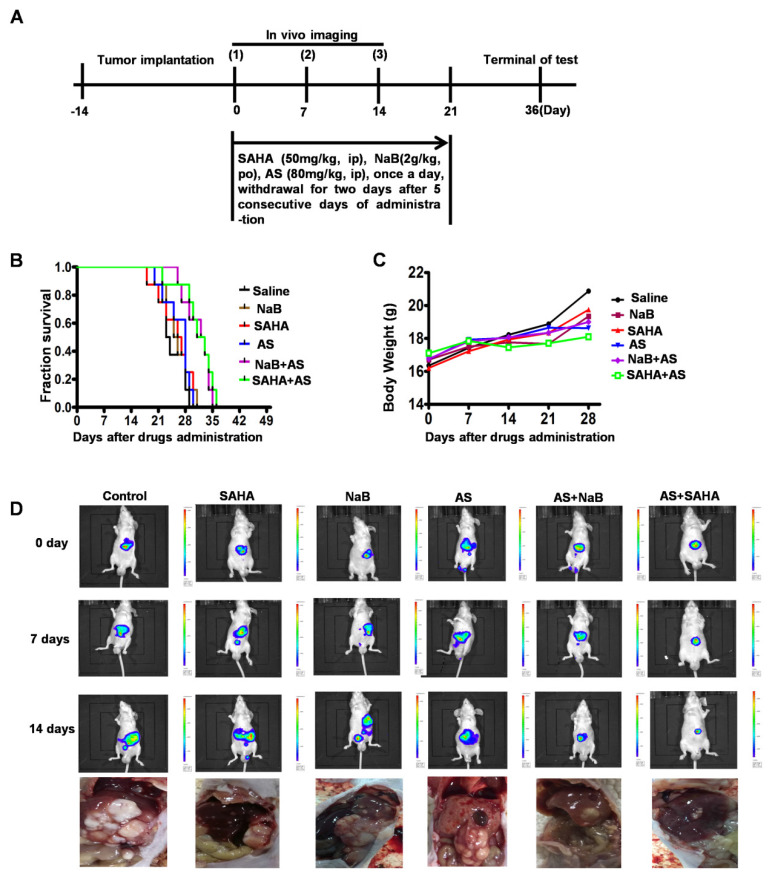
** HDIs combined with a FOXO1 inhibitor elicit a protective antitumor response in HepG2 tumor-bearing mice.** (**A**) Schematic representation of the treatment schedule and dosages in mice. (**B-D**) 5×10^6^ HepG2-luc cells were injected into the livers of nude mice with a 1 mL insulin injection needle, and the wounds were sutured with 5-0 silk thread. After 14 days, the mice were assigned to the following six groups, Saline, SAHA, NaB, AS, AS+SAHA, and AS+NaB. Mouse survival (B) and body weights (C) were measured at regular intervals; (D) The tumor volumes and metastasis were detected by *in vivo* imaging using luciferin. Representative images of metastatic liver tumors are shown.

We demonstrated that the FOXO1 inhibitor AS inhibited HDI-induced EMT and autophagy and enhanced cell apoptosis *in vitro* (Figures 4-6). To assess the efficacy of HDIs combined with the FOXO1 inhibitor AS *in vivo*, 5×10^6^ HepG2-luc cells were injected into the liver of nude mice. After 14 days, the mice were assigned to six groups, as illustrated in Figure 8A. Thirty days after drug administration, all mice treated with saline, SAHA, NaB or AS alone died, while the survival rate among mice treated with AS+SAHA or AS+NaB was about 50% (Figure 8B). The body weights of mice showed no significant differences among the six groups (Figure 8C). Furthermore, *in vivo* imaging demonstrated that mice treated with AS+SAHA (six of the eight mice) or AS+NaB (Five of the eight mice) had smaller tumor volumes than those in the other 4 groups, and metastasis occurred in mice treated with SAHA or NaB alone (Figure 8D). These results indicated that HDIs might promote metastasis by inducing EMT, an important contributor to tumor invasion and metastasis. However, the FOXO1 inhibitor AS could inhibit HDI-induced EMT and autophagy, and HDIs combined with a FOXO1 inhibitor elicited a synergistic pro-apoptotic effect.

To further investigate the antitumor mechanism of HDIs combined with the FOXO1 inhibitor AS *in vivo*, we performed immunohistochemical analysis to verify the expression of vimentin, E-cadherin, and Snail. It demonstrated that in HepG2 tumor tissues treated with HDIs, Snail and vimentin expression was significantly elevated compared with the control group, but when mice were treated with HDIs combined with AS, Snail and vimentin expression in tumor tissues was significantly reduced to levels close to that of the control group. In contrast, in mice treated with HDIs, E-cadherin expression was downregulated compared with controls, and when mice were treated with HDIs combined with AS, the E-cadherin expression in tumor tissue was elevated close to that of the control group (Figure S), demonstrating that HDIs could promote EMT and the FOXO1 inhibitor AS could inhibit HDI-induced EMT *in vivo*.

Next, we performed immunohistochemical studies to verify the expression of the autophagy-related markers FOXO1 and ULK1. As shown in figure S, in HepG2 tumor tissues treated with HDIs, FOXO1 and ULK1 expression was significantly elevated compared with the control group, but when mice were treated with HDIs combined with AS, FOXO1 and ULK1 expression in tumor tissue was significantly reduced to levels close to that of the control group (Figure S). These findings demonstrated that HDIs could promote the expression of the autophagy-related markers FOXO1 and ULK1, and the FOXO1 inhibitor AS inhibited HDI-induced expression of FOXO1 and ULK1 *in vivo*. These results demonstrated that HDIs combined with the FOXO1 inhibitor AS elicited a protective antitumor response in HepG2 tumor-bearing mice by inhibiting HDI-induced EMT and autophagy *in vivo*.

## Discussion

In HCC, aberrations in histone modifications, such as acetylation, have been shown to be important for tumor progression as well as prognosis and were proposed as a promising therapeutic target 25, 26. HDACs, the key components of the epigenetic machinery regulating histone acetylation and gene expression, play vital roles in tumor proliferation, invasion, and metastasis 27. Given their potent tumor-selective effects, inhibition of HDAC activity by HDIs is considered a promising novel therapeutic approach. Several HDIs, including SAHA, TSA, and romidepsin, are currently in clinical trials 28, 29. However, these inhibitors for the treatment of HCC as monotherapy has thus far demonstrated limited success. Also, considering the spectrum and the role of HDAC expression in the metastatic cascade, its inhibition may create undesirable effects 9. The mechanisms responsible for the limited clinical outcomes of the single-agent activity of HDIs on epithelial-derived cancer remain to be elucidated.

We previously demonstrated that HDIs triggered EMT in CNE2, LoVo, and HepG2 cells to promote invasion and metastasis by inducing the transcriptional expression of Snail via the Smad2/3 pathway 30. Furthermore, HDIs promoted acetylation of Snail, thereby inhibiting its ubiquitination and repressing its degradation 8. However, the impact of HDIs on EMT differs considerably in various types of cancers. In lung and breast cancers, most HDIs inhibited EMT with the exception of SAHA 31, 32. Similarly, in head and neck and prostate cancers all analyzed HDIs inhibited EMT 33,34. On the contrary, in hepatocellular carcinoma, most HDIs (VPA, SAHA, TSA, MS-275, NaB) stimulated EMT 8,35. Although this effect is cancer-type dependent, HDIs are considered as modifiers of the expression of EMT-related factors. In the present study, our results highlighted the importance of autophagy in HDI-induced invasion and metastasis. Significantly, we found that HDIs induced autophagy through the FOXO1-dependent pathway, and inhibition of FOXO1 sensitized hepatoma cells to HDIs and overcame autophagy-mediated metastasis.

Activation of compensatory pathways is frequently observed in response to various targeted therapies limiting their efficacy 36-38. Emerging evidence suggested that protein acetylation is involved in regulating autophagy 39, which functions as a pro-survival pathway during HDI-based chemotherapy 19, 40. Consistent with a previous report 41, we found an increase in autophagy in HCC upon treatment with HDIs that is likely an adaptive response to HDAC inhibition. This observation led us to consider whether HDI-induced HCC metastasis could be mediated by autophagy. Using RNA sequencing analysis, we found that FOXO1 and ULK1, two autophagy-related genes, were significantly upregulated in HDI-treated HCC cells, whereas the knockdown of FOXO1 or ULK1 blocked HDI-induced autophagy and EMT in hepatoma cells. These results suggested that the compensatory activation of autophagy-related genes upon HDAC inhibition triggers EMT, invasion, and metastasis of hepatoma cells. This notion may explain the limited efficacy of HDIs when used as monotherapy in patients and suggests that autophagy suppression combined with HDIs is a logical strategy.

Our study has afforded a novel mechanistic insight for HDI-induced metastasis mediated by the FOXO1-ULK1-Snail signaling axis. The FOXO1 transcription factor is considered a tumor suppressor because of its role in inhibiting cancer cell proliferation and inducing cell apoptosis 42. Unexpectedly, we identified a pro-metastasis function of FOXO1 in hepatoma cells by activating autophagy. Previous studies have established a critical role of FOXO1 in autophagy induction 43, 44. For example, activation of FOXO1 has been identified as a mediator of autophagy in chemically-induced oxidative stress 45. Our data are generally consistent with earlier reports that HDIs induce autophagy through FOXO1-dependent pathways. It has been shown that FOXO1 transcriptionally upregulates the expression of ATGs/Atgs 19. Our results also showed that FOXO1 activation by HDIs upregulated ULK1 expression in hepatoma cells, whereas HDI treatment failed to induce ULK1 expression after FOXO1 knockdown or inhibition. Moreover, our results suggested that the autophagy induction by FOXO1 was responsible for HDI-induced hepatoma cell metastasis since FOXO1 knockdown in hepatoma cells blocked HDIs-induced HCC cell autophagy, EMT, invasion, and migration.

After confirming the role of the FOXO1-autophagy pathway in HDI-mediated metastasis, we next investigated whether inhibition of FOXO1 potentiates the anti-cancer effects of HDCAIs in HCC. We found that individual treatment with FOXO1 inhibitors had only a slight suppressive effect on HCC proliferation and survival. However, a FOXO1 inhibitor exhibited a synergistic effect with HDIs in impeding cell growth *in vitro* and *in vivo*, indicating that the induction of autophagy by FOXO1 had a critical role in the development of intrinsic HDI resistance. Targeting FOXO1-mediated autophagy greatly enhanced the anti-proliferative and pro-apoptotic effects of HDIs.

In conclusion, our results suggest that HDIs trigger FOXO1-dependent autophagy in hepatoma cells that may ultimately limit the clinical outcomes of HDI-based therapies. Thus, the simultaneous blockade of the FOXO1-autophagy pathway with the use of HDIs might be an effective therapeutic strategy for the treatment of HCC.

## Supplementary Material

Supplementary figure.

## Abbreviations

EMT: Epithelial-mesenchymal transition
HCC: Hepatocellular carcinoma
FOXO1: Forkhead box O1
DAPI: 4,6-diamidino-2-phenylindole
HDAC: histone deacetylase
HDIs: Histone deacetylase inhibitors
TSA: Trichostatin A
NaB: Sodium butyrate
SAHA: Suberoylanilide hydroxamic acid
FITC: Fluorescein isothiocyanate
EMSA: Electrophoresis mobility shift assay
AMPK: Adenosine monophosphate-activated protein kinase
AS: FOXO1 inhibitor AS1842856
siRNA: small interference RNA
GAPDH: Glyceraldehyde 3-phosphate dehydrogenase
ULK1: Unc-51-like kinase 1
