# Supplementary material for: Histone deacetylase inhibitors promote epithelial-mesenchymal transition in Hepatocellular Carcinoma via AMPK-FOXO1-ULK1 signaling axis-mediated autophagy
Source: Theranostics. 2020 Aug 13;10(22):10245–61. doi: 10.7150/thno.47045 (PMC7481427; doi:10.7150/thno.47045)

Figure legends

Figrue S: The effect of HDIs combined with AS on expression of the markers of autophagy and EMT in vivo. Treatment schedule and dosages in mice as shown in figure 8A. 5×10^6 HepG2-luc cells were injected into the livers of nude mice with a 1 mL insulin injection needle, and the wounds were sutured with 5-0 silk thread. After 14 days, the mice were assigned to the following six groups, Saline, SAHA, NaB, AS, AS+SAHA, and AS+NaB, the animals were sacrificed, the mouse liver tissues were used for immunohistochemical staining to analyze the expression of vimentin, Snail, E-cadherin, FOXO1, and ULK1, scale bar, 200 μm.

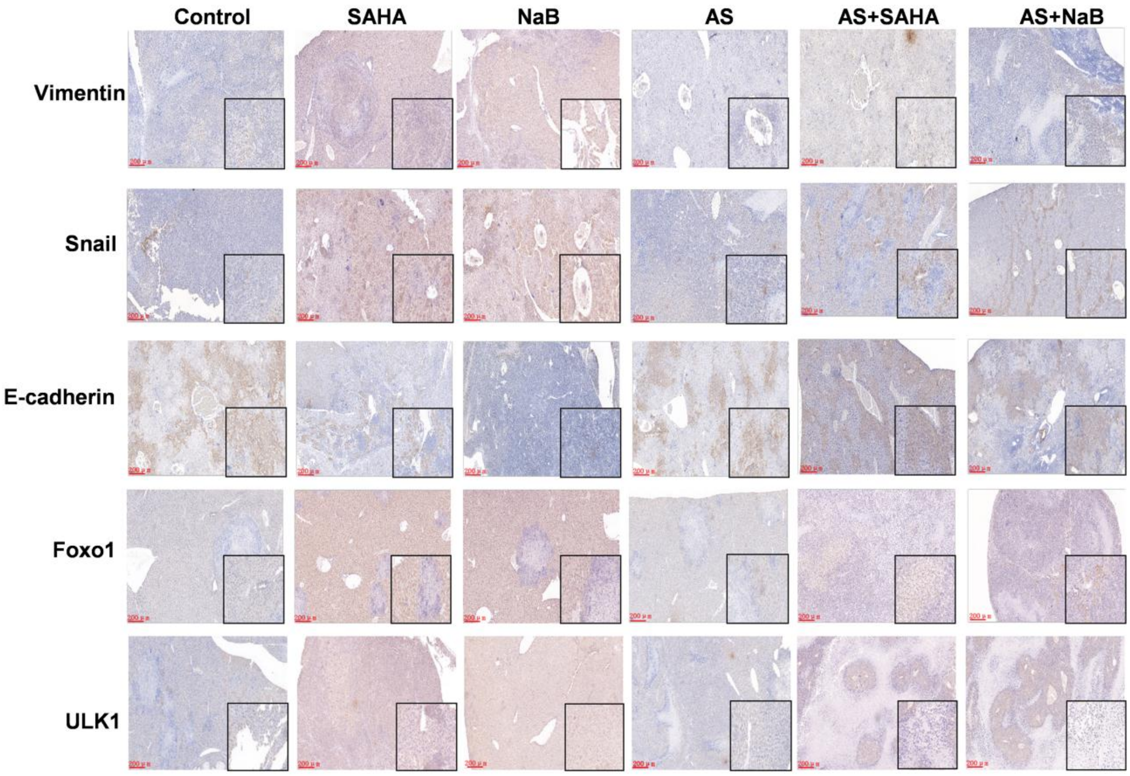

Supplement: Supplementary file 1 — Supplementary figure. [file thnov10p10245s1.pdf]
